# Perception is Only Real When Shared: A Mathematical Model for Collaborative Shared Perception in Human-Robot Interaction

**DOI:** 10.3389/frobt.2022.733954

**Published:** 2022-06-15

**Authors:** Marco Matarese, Francesco Rea, Alessandra Sciutti

**Affiliations:** ^1^ DIBRIS Department, University of Genoa, Genoa, Italy; ^2^ RBCS Unit, Italian Institute of Technology, Genoa, Italy; ^3^ CONTACT Unit, Italian Institute of Technology, Genoa, Italy

**Keywords:** shared perception, human-robot interaction, theory of mind, joint attention, shared autonomy, nonverbal communication, gaze cues

## Abstract

Partners have to build a shared understanding of their environment in everyday collaborative tasks by aligning their perceptions and establishing a common ground. This is one of the aims of shared perception: revealing characteristics of the individual perception to others with whom we share the same environment. In this regard, social cognitive processes, such as joint attention and perspective-taking, form a shared perception. From a Human-Robot Interaction (HRI) perspective, robots would benefit from the ability to establish shared perception with humans and a common understanding of the environment with their partners. In this work, we wanted to assess whether a robot, considering the differences in perception between itself and its partner, could be more effective in its helping role and to what extent this improves task completion and the interaction experience. For this purpose, we designed a mathematical model for a collaborative shared perception that aims to maximise the collaborators’ knowledge of the environment when there are asymmetries in perception. Moreover, we instantiated and tested our model *via* a real HRI scenario. The experiment consisted of a cooperative game in which participants had to build towers of Lego bricks, while the robot took the role of a suggester. In particular, we conducted experiments using two different robot behaviours. In one condition, based on shared perception, the robot gave suggestions by considering the partners’ point of view and using its inference about their common ground to select the most informative hint. In the other condition, the robot just indicated the brick that would have yielded a higher score from its individual perspective. The adoption of shared perception in the selection of suggestions led to better performances in all the instances of the game where the visual information was not *a priori* common to both agents. However, the subjective evaluation of the robot’s behaviour did not change between conditions.

## 1 Introduction

The ability to cooperate and communicate is inherent in human nature. People can easily share information with others to achieve common objectives. However, human interactions require a common ground to succeed in reaching a shared goal (Thomaz et al., 2019). The lack of this common ground could cause misunderstanding and mistakes even in simple collaborative tasks, e.g., when two agents perceive the same objects differently (Chai et al., 2014). Indeed, between collaborating agents, even a slight misalignment on the common ground may be due to different perceptions of the shared environment. Each agent can have a peculiar perception of such an environment because of differences in perspective, sensory capabilities (e.g., colour-blindness) or prior knowledge (Mazzola et al., 2020).

Despite the different perceptions of a shared environment, people can naturally interact with each other. Two collaborators can align on a common ground of beliefs, intentions and perceptions, ideally maximising both performances and the shared knowledge. Establishing shared perception aims to build a common understanding of the environment by bridging the different individual perceptions. For instance, this implies revealing what is hidden in the eyes of a collaborator, annulling perceptual asymmetries. Even when this is not entirely possible, e.g., the hidden item cannot be uncovered, a partner can leverage the shared knowledge to reveal something about covered items to help the collaborator make informed actions. Moreover, suppose the two partners have a good understanding of the other’s goals and intentions. In that case, they will also know how to maximise the shared knowledge, selecting when it is crucial to reveal the differences in individual information and when it is more effective to focus only on the shared space.

A crucial aspect of shared perception is perspective-taking (Wolgast et al., 2020). By taking the point of view of a partner, an agent can understand the differences between their perception and that of their collaborators. Furthermore, the ability to understand partners’ actions as guided by intentional behaviours and to ascribe to them mental states is called Theory of Mind (ToM) (Görür et al., 2017). Shared perception is a pivotal part of ToM because, by combining it with perspective-taking, an agent can more easily infer the rationale behind one’s actions and - more importantly - understand that a collaborator’s unreasonable action could be due to a mismatch between their perceptions.

From the robotic point of view, shared perception can help robots infer their partner’s intentions and, taking their point of view, reason over them and consequently select the most effective collaborative action. By providing help in establishing a common understanding of the shared environment between the two partners, shared perception is particularly beneficial when human and robotic perceptions are not identical. This might happen in scenarios where the robot can perceive advantageous characteristics of the environment that the human collaborator can not. In particular, considering a one-to-one Human-Robot Interaction (HRI) scenario, both the human and robot could benefit from building a ToM of the other and establishing a shared perception. Considering ToM, the human needs to translate the robot’s actions in terms of objectives, beliefs and intentions (Scassellati, 2002), while the robot needs to infer its collaborator’s mental states (Devin and Alami, 2016) to anticipate the unfolding of the following actions better. Shared perception is then fundamental to allow each of the two partners to be aware of what the other can perceive and which action should be performed to maximise the potential of achieving such objectives.

Let us imagine a person assembling an Ikea piece of furniture with their domestic robot. This task needs some tools (e.g., screwdrivers, screws, bolts) to assemble the different parts (e.g., shelves). Each tool has different characteristics that make it useful or useless to assemble a particular part of the piece of furniture. The assembling task, *per se*, can make the environment very chaotic, given all the building material. This brings possible obstacles in the person’s perceptual space and could result in asymmetries in the perception between the two agents. In this setting, the robot can exploit shared perception by trying to resolve such asymmetries, verbally communicating the characteristics of potentially valuable objects, or handing over the object which is the best according to what it sees and what it infers about what the human partner is seeing.

The field of HRI has dedicated wide attention to the social phenomena that constitute the backbone of shared perception, such as joint attention (Nagai et al., 2003), perspective-taking (Fischer and Demiris, 2016), common knowledge (Kiesler, 2005), communication (Mavridis, 2015) and ToM (Bianco and Ognibene, 2019). In this work, we wanted to investigate how these mechanisms work in synergy to lead to shared perception between a human and a robot and what impact shared perception has on HRI.

**FIGURE 1:**
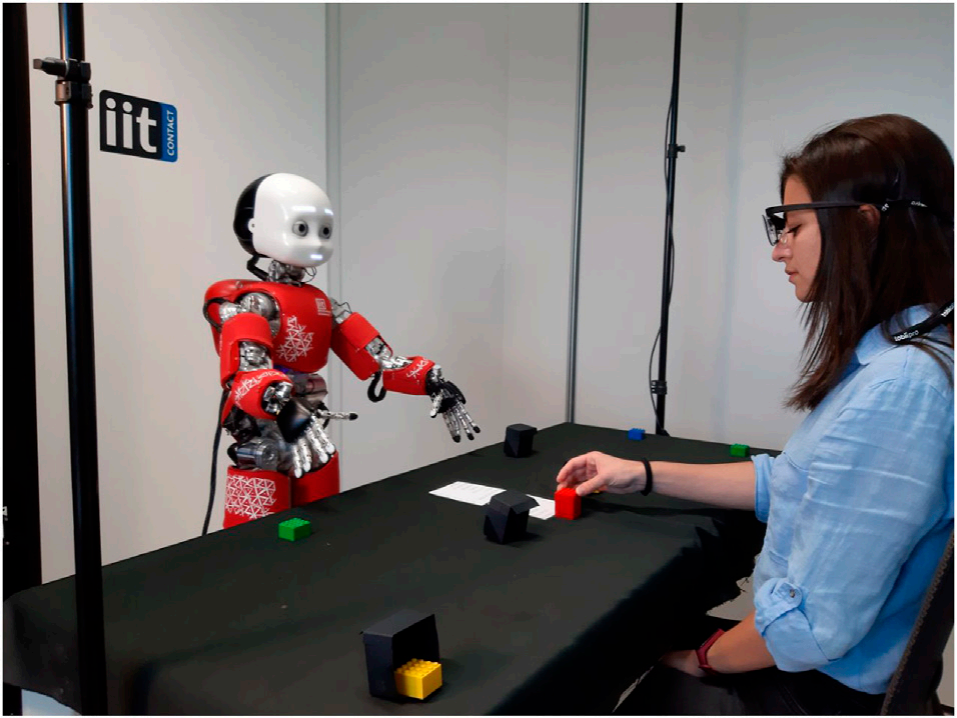
A participant and the robot iCub performing the task.

Hence, we present a mathematical model for shared perception, through which a collaborative robot aims at maximising both performances and the collaborator’s knowledge about the environment. To test the model, we asked participants to play a cooperative game with the iCub robot in a real HRI scenario in which they had to build a tower with LEGO bricks (Figure 1). During the task, the robot could either leverage shared perception principles (SP) or just aim at maximising the overall task performance (NSP). We designed our experiments to create specific critical moments—the *conflicts*—characterised by a mismatch between participants’ and robot’s perceptions.

The following sections are organised as follows: Section 2 presents an overview of related works; Section 3 describes the mathematical model, the experiment and the software architecture; Section 4 concerns the experimental results. The last two sections are dedicated to discussion and conclusion.

## 2 Background and Related Works

Shared perception is a complex mechanism, that entails a range of social skills. A robot, to establish shared perception, would need the awareness that the collaborator could have a different perception and should also be aware of which are those differences, e.g., what parts of their perceivable environment are hidden to its partner. Furthermore, it would need to know the other’s goal and its relation to the objects in the environment. Last, the robot should estimate how the partner understands its own behaviour to provide communications that the collaborator can effectively understand and enact.

One of the fundamental mechanisms of the understanding that others might perceive the world differently is Perspective-Taking (PT). PT is *“a multifaceted skill set, involving the disposition, motivation, and contextual attempts to consider and understand other individuals”* (Wolgast et al., 2020). As well as humans, a robot can infer humans’ perception through mechanisms of PT: it has been proved that PT also improves action recognition performances (Johnson and Demiris, 2005; Johnson and Demiris, 2007). Therefore, algorithms for PT in HRI have been proposed to disambiguate whether an object is visible to people facing the robot, using just their head pose (Fischer and Demiris, 2016). Moreover, several PT-based architectures have been proposed to estimate where a person will execute a future task (Pandey et al., 2013), to then produce proactive and collaborative behaviours. Other contexts in which PT has been investigated are the military field (Kennedy et al., 2007), where the robot used those mechanisms to understand if it was visible to an enemy or in human-robot teaching scenarios (Berlin et al., 2006; Breazeal et al., 2006). Several works showed that PT is beneficial to disambiguate both things and circumstances (Ros et al., 2010), such as tools and commands (Trafton et al., 2005a; Trafton et al., 2005b). Hence, we can use PT mechanisms to “put ourselves in one’s shoes” so that we can understand their point of view and build a common ground on which to base an efficient collaboration (Brown-Schmidt and Heller, 2018). In this study, we focus on visual PT, which is the ability to see the world from another person’s perspective, taking into account what they see and how they see it (Flavell, 1977).

To better align the perspectives of two or more collaborating agents, we need that all of them build a reliable Theory of Mind (ToM) of the others (Marchetti et al., 2018). This means that, in addition to perception, partners have to base their interaction also on shared knowledge: at least, they need to know what the other agents already know so that they can easily anticipate others’ actions (Winfield, 2018). Several works in robotics and HRI took inspiration from other fields such as psychology and philosophy to model ToMs for robots. For example, Scassellati, (2002) discuss the theories presented in (Baron-Cohen, 1997) and (Leslie et al., 1994) on developmental ToM in children to build robots with similar capabilities. Rather, more recent works implement ToMs through a Bayesian model (Lee et al., 2019) to best solve human-robot nonverbal communication issues. In HRI, it has been shown that people appreciate robots that show ToM-like abilities as teammates for their ability to identify the most likely cause of others’ behaviour (Hiatt et al., 2011). This is also because people perceive such robots as more capable of aligning themselves to persons by fully recognising their environment (Benninghoff et al., 2013). Moreover, developmental human-inspired ToMs have been presented to enhance the quality of the HRI itself: e.g., in (Vinanzi et al., 2019), the authors modelled the trustworthiness of the robot’s human collaborator using a probabilistic ToM and a trust model supported by an episodic memory system.

The gaze plays a pivotal role in facilitating the understanding of others’ goals and enabling intuitive collaboration. Gaze movements have been proved to be very helpful in collaborative scenarios (Fischer et al., 2015). Pierno et al. (2006) observed the same neural response in people observing someone cueing an object and in people observing someone reaching an object to grasp it: gaze cues are a powerful indicator of people’s intentions. People are sensitive also to robot gazing when this signal is directed at an object in the environment. It has already been proved that people predict which objects to select using referential gaze cues from robots, even if they are not consciously aware of those cues (Mutlu et al., 2009). Indeed, through gaze cues, a robot could highlight parts of the environment, thus providing information about its perception (Fussell et al., 2003). Several studies proved that people are very good at identifying the target of partners’ referential gaze to use this information to predict their future actions (Staudte and Crocker, 2011; Boucher et al., 2012). When people refer to objects around them, they look at those objects before manipulating them (Griffin and Bock, 2000; Hanna and Brennan, 2007; Yu et al., 2012) and when partners refer to objects, people use their gaze to predict their following intentions to quickly respond to the partner’s reference (Boucher et al., 2012). In fact, with little information about the partner’s gaze, people are slower at responding to their partner’s communication (Boucher et al., 2012). Moreover, objects that are not related to a task are rarely fixated (Hayhoe and Ballard, 2005). In sum, beyond implicitly revealing an agent’s future intentions, gaze movements can be an effective form of nonverbal communication (Rea et al., 2016; Admoni and Scassellati, 2017; Wallkotter et al., 2021).

So far, the used approach in PT studies focused on the disambiguation of tools and commands to help the artificial agents build a common ground with their collaborators. With the current work, we want to move forward in using such mechanisms by considering robots capable of sharing information gathered from their own perspective but communicated by taking into consideration both the perspective of their human partners and the shared knowledge. Through shared perception mechanisms, we aim to go further in this approach by building a model that can enable robots to autonomously resolve situations characterised by asymmetries between their perception and one of their collaborators.

In the current work, contrary to what is already present in the literature about asymmetries in perception in HRI (Chai et al., 2014), we underline how the issue of creating a common ground also applies to interactions not mediated by language. Even in scenarios where the action possibilities are constrained, and the goal of the task is clear, the mismatch in perception requires a communicative effort to establish a shared understanding. We show that this can also be achieved with non-verbal signals.

For this purpose, in this paper, we provide a mathematical model that supports shared perception-based decisions for a robot helper in a collaborative task. We assess task performance and interaction experience when this model guides the robot hints. We compare them with interactions in which robot behaviour is driven just by the goal of maximising the task score.

## 3 Materials and Methods

### 3.1 The Mathematical Model

The mathematical model for collaborative Shared Perception (SP) adopts a formulation taken from the theory of sets and probability. The elements that characterise the model are presented in a general and abstract way so that they can be instantiated depending on the circumstances. In particular, the model manipulates concepts such as objects, environment, personal/common perception and awareness. Here, we do not provide examples of instantiating those concepts, but later in this section, we discuss how we did it for our experiment.

The model considers only one-to-one interaction; thus, we always have an agent (*a*_1_) aiming to share their perception with another agent (*a*_2_). In this work, we consider *a*_1_ as a robot and *a*_2_ as a person. The model’s core is a sort of common knowledge between the two agents that we call “common awareness”. In particular, *a*_1_ exploits elements belonging to this common knowledge to give insights about elements in its individual perception. Hence, the model’s objective is to enable robots to exploit SP mechanisms. For this purpose, it aims to maximise partners’ awareness of objects they can not perceive by using elements belonging to the common ground already established. In particular, the model tries to share its individual perception with the partner, choosing the elements of common awareness that it could most appropriately exploit. In this sense, our model is about decision-making and not just communication.

To present our model, we need first to define its elements. We define the **environment** *X* = {*x*: *x*is an object} as a finite set of objects. Thus, we adopt a *closed world* formulation: everything we consider belongs to the environment.

Moreover, we define an **object** *x* ∈ *X* as a finite set of characteristics, as follows: *x* = {*c*_1_, … , *c*_*n*_: *c*_*i*_is a characteristic*∀i* = 1, … , *n*} where, for *characteristics*, we mean features such as colour, shape, etc. An object’s characteristic have to be instantiated, *i.e.* if *c*_1_ refers to the object’s colour, we could have *x* = {*c*_1_ = *blue*, … }.

Moreover, an agent couples all these characteristics with a probability distribution describing the agent’s degree of certainty on each characteristic’s instance. Thus, from the agent *a*’s point of view, the object *x* is a set of pairs where, to each object’s characteristic, it is associated with a probability distribution over the set of all its possible instances: $x=\left\{\left(c_{1},{\mathrm{Pr}}_{a}^{x}\right),\ldots ,\left(c_{n},{\mathrm{Pr}}_{a}^{x}\right)\right\}$ . The probability distribution functions associated with the objects’ characteristics can be derived from the task rules if the collaborative task is constrained. For example, the agent *a* associates to the characteristic “colour,” let us say *c*_1_, of the object *x* a probability distribution ${\mathrm{Pr}}_{a}^{x}$ over the set of all the possible instances, let us say *blue*, *red* and *black*. Assuming that *x* is *blue*, if *a* knows that *x* is blue, then we would have ${\mathrm{Pr}}_{a}^{x}\left(c_{1}=blue\right)=1$ ; on the other hand, if *a* has no information about the colour of *x*, then we would have ${\mathrm{Pr}}_{a}^{x}\left(c_{1}=blue\right)=0.33$ , ${\mathrm{Pr}}_{a}^{x}\left(c_{1}=red\right)=0.33$ , and ${\mathrm{Pr}}_{a}^{x}\left(c_{1}=black\right)=0.33$ . The implicit assumption is that the objects belonging to the environment do not change over time.

With the elements described above, we can define the **Personal Perception** of the agent *a*, *P*_*a*_ = {*x* ∈ *X*: the agent*a*can perceive*x*} as the finite set of the objects belonging to *a*’s perception. From the definition of *P*_*a*_ follows that *P*_*a*_ ⊆ *X* for each agent *a*. The definition of personal perception depends on the agent’s capability: *i.e.*, if the agent *a* is a robot equipped with only a camera, then *P*_*a*_ refers to the object the robot can see through its camera.

Similarly, we define the **Awareness Space** of the agent *a*, *W*_*a*_ = {*x* ∈ *X*: *a*is aware that*x* ∈ *X*} as the finite set containing the objects of which *a* is aware. We characterise the set *W*_*a*_ as follows: *∀x* ∈ *X*, if *x* ∈ *P*_*a*_⇒*x* ∈ *W*_*a*_ for each agent *a*. Thus, it follows that *P*_*a*_ ⊆ *W*_*a*_ ⊆ *X* for each agent *a*.

We say that the agents *a*_1_ and *a*_2_ both perceive the object *x* if *∃x* ∈ *X*: $x\in P_{a_{1}}$ and $x\in P_{a_{2}}$ . As well, we say that the agents *a*_1_ and *a*_2_ are both aware of the object *x* if *∃x* ∈ *X*: $x\in W_{a_{1}}$ and $x\in W_{a_{2}}$ . Thus, we define the **Common Perception** between the agents *a*_1_ and *a*_2_ as follows: $P_{a_{1},a_{2}}^{c}=P_{a_{1}}\cap P_{a_{2}}$ . Similarly, we define the **Common Awareness** between *a*_1_ and *a*_2_ as follows: $W_{a_{1},a_{2}}^{c}=W_{a_{1}}\cap W_{a_{2}}$ . Hence, from the characterisation of *W*, we have that if $x\in P_{a_{1},a_{2}}^{c}\Rightarrow x\in W_{a_{1},a_{2}}^{c}$ .

A SP communication from the agent *a*_1_ to the agent *a*_2_ $\left(a_{1}\overset{\text{SP}}{\to }a_{2}\right)$ aims to maximise the knowledge of *a*_2_ of the objects belonging to the personal perception of *a*_1_, *P*_*a*_, that do not belong to the awareness space of *a*_2_, $W_{a_{2}}$ . To achieve this objective, SP exploits the common characteristics between objects belonging to *a*_1_’s personal perception and those belonging to *a*_1_ and *a*_2_’s common awareness. Thus, we say that the SP is possible if the following condition occurs:

$\left\{\begin{array}{c} \exists x_{1}\in P_{a_{1}}\text{ so that }x_{1}=\left\{c,c_{m},\ldots ,c_{n}\right\}\;\\ \exists x_{2}\in W_{a_{1},a_{2}}^{c}\text{ so that }x_{2}=\left\{c,c_{l},\ldots ,c_{t}\right\}\; \end{array}\right.$

(where m,n,l,t∈N ). In other words, if the object *x*_1_, belonging to the personal perception of *a*_1_, shares at least one characteristic with the object *x*_2_, belonging to the common awareness of the two agents, then it is possible to communicate such common characteristics to give insights about *x*_1_. If this precondition is respected, we have that $\exists f:W_{a_{1}}\mapsto W_{a_{2}}$ and $\exists {\hat{x}}_{1}\in W_{a_{2}}$ so that $f\left(x_{1}\right)={\hat{x}}_{1}$ : $c\in {\hat{x}}_{1}$ . It means that *x*_1_ is approximated by *a*_2_ with the object ${\hat{x}}_{1}$ that contains the characteristic *c* that *a*_1_ shared through its communication. The goals of the task influence the target of the communication. In the current model, we assume that such objectives are common between the two agents because of their collaboration.

The objective of the SP is to maximise ${\mathrm{Pr}}_{a_{2}}^{x}\left(c=actual\text{\_}c\text{\_}value\right)$ , thus to minimise *a*_2_’s degree of uncertainty about such a characteristic. In the best case, when this uncertainty becomes zero because of *a*_1_’s communication, *a*_2_ can be sure of the value of *c*: it means that ${\mathrm{Pr}}_{a}^{x}\left(c=actual\text{\_}c\text{\_}value\right)=1$ . This way *a*_1_ makes ${\hat{x}}_{1}$ less and less approximated and, once *a*_1_ can communicate all *x*_1_’s characteristics, or once *a*_2_ can infer all of them, we have ${\hat{x}}_{1}=x_{1}\Rightarrow x_{1}\in W_{a_{2}}\Rightarrow x_{1}\in W_{a_{1},a_{2}}^{c}$ . However, it is not always possible to make $\hat{x}$ collapse on *x* because it depends on both the agent’s communication capabilities and context. In general, the objective of the shared perception $a_{1}\overset{\text{SP}}{\to }a_{2}$ on the object *x* is:

*∀c*_*i*_ ∈ *x*, $\max{\mathrm{Pr}}_{a_{2}}^{x}\left(c_{i}=actual\text{\_}c_{i}\text{\_}value\right)$ .

In most cases, it is not necessary to cancel the uncertainty or communicate all the object’s features. Most importantly, we need to communicate the essential characteristics for the goal and improve the probability of having the right information so that the partner can make the right decision. From our formulation, follows that if $∄x\in W_{a_{1},a_{2}}^{c}\Rightarrow$ it is not possible to do shared perception.

We point out that one of the main preconditions for a good functioning of the model is that both the robot and human are capable of using recursive ToM (Woodruff and Premack, 1978; Arslan et al., 2012). In short, recursive (or second-order) ToM is the ability to reason over the others’ estimation of our own mental states. People are good at using recursive ToM with their peers (*e.g.*, in strategic games (Goodie et al., 2012)) and recently de Weerd et al. (2017) proved that they spontaneously use recursive ToM also with artificial agents when these latter are capable of second-order ToM as well. Without recursive ToM, the robot could not assume that the human is aware of its own knowledge about the partner’s difference in perception. At the same time, the human could not make sense of a robot’s suggestion, without the awareness that the robot has a model of what that person perceives. Hence, the proposed model would not be effective without recursive ToM, as the selection of the most informative characteristic, and of how to communicate it, rests on the assumption that both agents entertain such understanding of each other. More in general, without recursive ToM a robot could not exploit at its best the everyday awareness it builds with its human collaborator. Moreover, both the robot and human would be weakly aligned (or, even worse, not aligned at all) on beliefs, desires and intentions.

### 3.2 The Experiment

We asked participants to build a tower with a maximum of five LEGO bricks by picking them among the ones available on the table in front of them. The bricks had different colours: we associated a score with each colour (Table 1). Participants could put a brick on the tower’s top on each round, but only if its value was less or equal to the brick previously on top. The game ended when the tower was complete; i.e., either after five rounds or when all the available bricks had a higher value than the one on the top. The goal of the game was to maximise the score of the tower. The experimenter explained the rules before task initiation, underlining the importance of maximising the score of the tower.

**TABLE 1:** The list of colours, and their values, that participants could consult during the experiments.

Colour	Value
Pink	3
Orange	4
Blue	4
Yellow	6
Black	6
Green	8
White	10
Red	10

Figure 1 shows the experimental setup. Both the participants and iCub sat at a table, facing each other during the experiment. On the table, there was a sheet of paper reporting the values of the colours (Table 1), eight coloured bricks and four *obstacles*. The obstacles were little constructions that could hide a brick. Because of these, the iCub could not perceive two bricks, while the participants could not perceive the other two. Then, there were other four bricks that both iCub and the participants could see.

The task was presented as a collaborative game with the robot. The participants were the *builders*: they have to physically take one brick at a time from the table and insert it on the top of the tower. Instead, the robot was the *suggester*: it could suggest which brick to take.

Each round of the game was structured in three distinct phases: i) the inspection, ii) the communication and iii) the action. The inspection phase i) had a fixed duration of 15 seconds. During this period, the participants should inspect the table to choose a candidate brick to put on the tower during the action phase. In the communication phase ii), the robot provided its suggestion by looking at the brick it wanted to propose to the partner. We told participants that, during the second phase, they would expect a suggestion from the robot, which was collaborating with them. Three seconds after the robot’s gaze motion, an acoustic signal informed the participants of the beginning of the action phase iii). In this latter phase, the participant chose, picked up a brick and positioned it on the tower. At the beginning of this phase, iCub stared at the tower under construction. Once the participants placed the brick on the tower, the robot inspected the table with its gaze. We limited the communication between the participants and the robot to keep the interaction as minimalist as possible: indeed, we allowed them to use only gaze.

**FIGURE 2:**
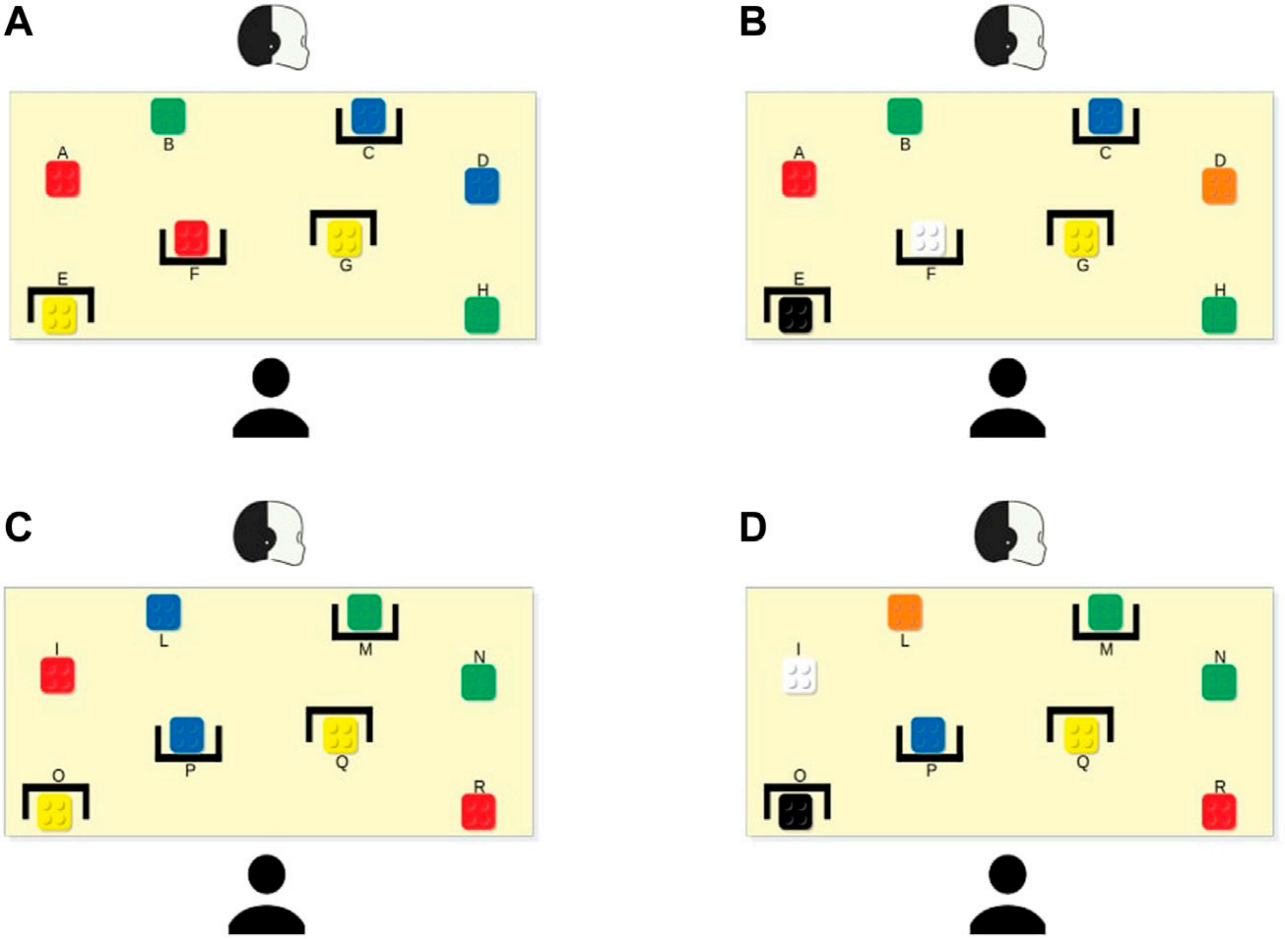
The four bricks’ setups used in the experiments. Setups **(A,B)** and **(C,D)** are equivalent, respectively, meaning that, even if it seems that they are different, they include bricks with the same value in the same position (red and white bricks, and black and yellow ones have the same value).

We choose the bricks’ configurations to force critical moments (*conflicts*), where the selection of the best brick was different considering the personal views of the two agents. We ran a series of task simulations to find the configurations that maximised such differences. Once found, we selected two; then, we created the other two configurations by replacing some bricks with ones of a different colour but equal value. Thus, the participants had the feeling of playing with four different configurations; however, they played with only two configurations. This way, each participant could perform the task with both robot’s modes (SP and NSP—see description below) in both bricks’ configurations. This simple expedient allowed us to present the same conflicts, for each condition, to all participants. In particular, we performed a within-subject user study so that each participant could face both the experimental conditions. Figure 2 shows the configurations we used: the configuration in Figure 2A was equivalent to the one shown in Figure 2B; the same applies to Figure 2C and Figure 2D.

Participants did not know *a priori* which sets of brick colours were present in each session. In addition, Table 1 also shows pink as a possible brick colour, corresponding to the lowest value of all, but none of the configurations had pink bricks. We added such a colour to avoid settings where participants could know *a priori* that the bricks visible to them corresponded to an overall minimum value.

Before each session, we told the participants that they would interact with a robot powered by a new program, so it would be like interacting with a different robot. In both conditions, the robot had the same knowledge of the environment—the position of the bricks it could see (i.e., not occluded to it) and their colour—and it used the exact internal representation of the task. Regardless of the robot mode, iCub always suggested one of the most valuable bricks to maximise the tower’s value.

The difference between SP and NSP behaviours was in the order of the suggestions in case of multiple best options from the robot’s perspective (e.g., during the conflicts). The SP-iCub, following the shared perception model, hinted at the bricks which would maximise the information about the relevant properties of the bricks hidden to participants. On the other hand, the NSP-robot suggested the bricks following its internal representation of the task (see Section 3.2.1.1), without taking into account the perspective of its partner. In the current experiment, such internal representation actually led the robot to behave in the conflicts in opposite ways in the SP and NSP settings. This way, we ensured the maximum difference between the two robotic behaviours.

All the participants had to perform four trials, one for each configuration of the bricks: during one session of two consecutive trials, they had to interact with the SP-robot and, during the other two consecutive trials, with the NSP-robot. We counterbalanced the order of the presented setups and the robot mode.

Before and after each experiment, we asked participants how many bricks they thought the robot could see, how many they could see, and how many bricks they thought were on the table. All participants answered that there were eight bricks on the table (during the instruction, we told them that there was a brick behind each obstacle), that they could see six of them, and that also iCub could see just six bricks. Thus, at both the beginning and the end of the experiments, participants understood that iCub could not see certain bricks. In our setup, we assumed that participants would accept the robot’s suggestions as the best options from its point of view. As we ensured after the experiments, all participants perceived the robot’s gazing as suggestions to take those bricks; they also reported to us that they assumed it behaved like that based on the colour of the bricks.

Familiar situations that reflect the mechanisms of our task are competitive team card games. In a card game, each player has both private individual perception (the cards in their hand) and a common perception with the others (the cards on the board). In team-based games, people in the same team aim to maximise both the chances of victory and partners’ knowledge of the cards in their hands. We want to take as an example the game of *“Briscola”* ^1^: a famous Italian competitive turn-based card game involving two teams, each consisting of two players. Here, players have to discard one card *per* turn, and both value and seed of the cards determine which team scores points. During the game, the partners try to inform each other of their private cards by strategically selecting which cards to discard. The game rules forbid players to inform the partner about the cards in their hand directly; thus, they have to play aiming at maximising both the probability of winning and the possibility for the partner to infer their cards. In our experiment, the SP-robot acted with this double aim, while the NSP-robot played only with the first objective.

#### 3.2.1 The Experimental Software Architecture

To perform our experiment, we developed a software architecture composed of three main modules: the knowledge module, the communication module, and the reasoner.

##### 3.2.1.1 The Knowledge Module

The knowledge module aimed to manage the knowledge base; also, it provided information to the reasoner. The knowledge base was defined once at the beginning of the experiment and updated online after each move. It maintained a graph to represent the task and a stack to represent the available bricks. The stack stored the bricks in decreasing value order to consider them in such decreasing order: it maintained only the bricks that the robot could perceive. In the stack, the bricks with the same value respected their positioning order on the table. Moreover, through the graph, the robot could track the task’s progress and the next possible moves. The graph’s vertices represented the bricks on the table, while the arrows represented the possible moves: the vertex *i* had an arrow towards the vertex *j* if and only if it could stack the brick *j* after the brick *i*. According to participants’ choices, both the graph and the stack were updated during the task.

##### 3.2.1.2 The Communication Module

The communication module mainly aimed at sending commands to a control module that accounted for both robot’s neck and eyes kinematics: the *iKinGazeCtrl* module (Roncone et al., 2016). It combines those independent controls to ensure the convergence of the robot’s fixation point on its target. The *iKinGazeCtrl* module allowed the robot to have biologically inspired movements: this makes the robot’s movements more natural. We used a combined approach because eye-based estimation of the observed location has been proven to be much more informative than head-based one, at least for human observations (Palinko et al., 2016).

##### 3.2.1.3 The Reasoner

The Reasoner aimed to guide robot behaviour by collecting information from the Knowledge Module, reasoning over them and then deciding the robot’s actions. In particular, it gathered from the Knowledge Module information about the possible next moves, the available bricks on the table, and what bricks were visible from its own perspective. We provided *a priori* this information to the robot. By exploiting this information, the reasoner knew what brick the robot should indicate to participants at each moment of the task.

In particular, in NSP mode, the reasoner used the following heuristic: it indicated the brick currently on the top of the knowledge base’s stack (according to the task’s rules). Such a brick was always one of the bricks with the higher value among those the robot could perceive (compared to the one currently on the top of the tower, since we designed the reasoner so that it would follow the game rules). Thus, in NSP mode, the robot considered only its perspective and task rules.

On the other hand, in SP mode, the reasoner had a more complex approach, also considering the collaborator’s perspective. Instead of considering only the brick on the top of the knowledge base’s stack—the *candidate* brick—it also reasoned on the bricks perceivable by both the robot and participants. If the second brick on the stack had the same value as the one on the top, then a conflict may have occurred: thus, the reasoner asked for information from the knowledge base to understand that. If it was not the case, the reasoner decided to behave as in NSP mode. In the former case, the reasoner picked the brick to indicate based on the SP model described in Section 3.1, aiming at minimising participants’ uncertainty about the relevant properties of the hidden brick.

#### 3.2.2 Conflicts

We designed our experiments to elicit two critical moments for each task: the *conflicts*. During the conflicts, there was a mismatch between participants’ perception and the robot’s since the best brick was hidden from the robot’s view or the participants’. This mismatch could lead to different brick choices because some important information was unavailable to one of the two agents involved. We designed three types of conflict: the *Main Conflict*, the *First Brick Conflict*, and the *Mid-Game Conflict*.

##### 3.2.2.1 The Main Conflict

**FIGURE 3:**
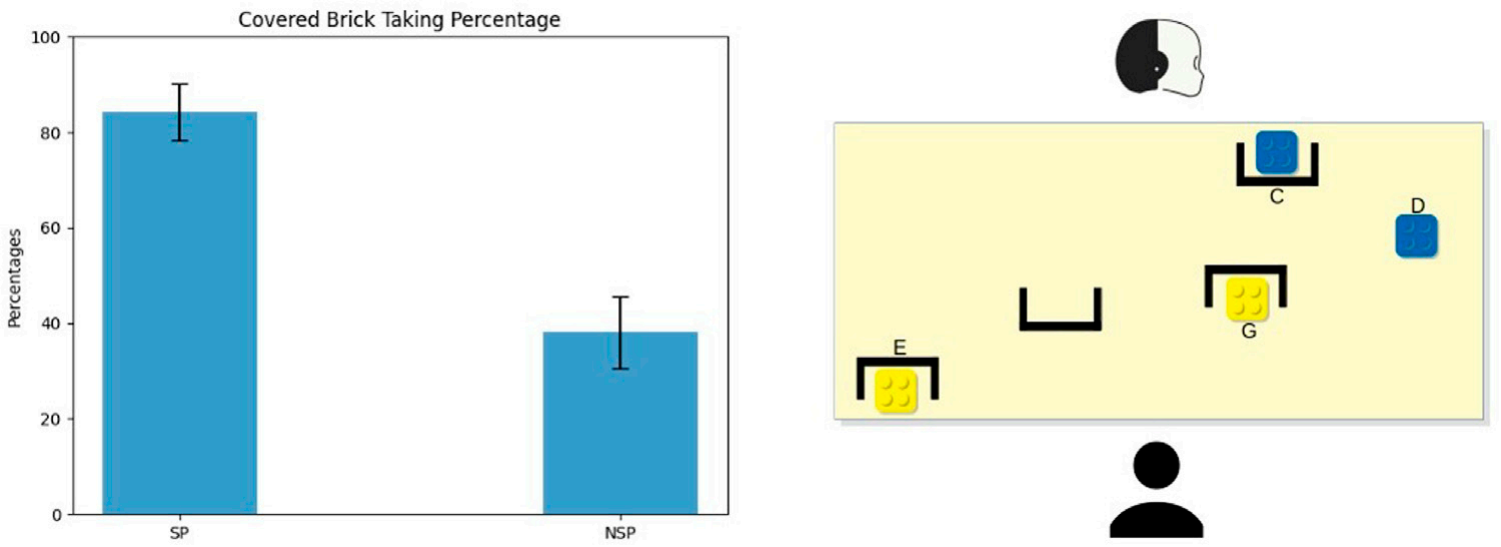
Average and standard error of the Main Conflict’s covered brick taking percentage. In this case, the covered bricks were the E and G ones.

The main conflict occurred in both the setups toward the end of the task: participants faced this type of conflict during each session, for a total amount of four main conflicts *per* participant. Figure 3 (right) shows an example of the bricks configuration during this conflict. Since this conflict was practically identical in both of the setups, here we present only the configuration of the first one.

The main conflict presented a situation where the robot could not see the best choice (the yellow bricks, G and E), which were instead visible to participants. At the same time, only the robot could see what lay behind the occlusion (C, the blue brick). This represented the second-best choice for the participant but one of the best choices in the robot’s view (together with the other blue brick D, visible to the participant as well).

This conflict yielded two different hints for the SP-based robot and the NSP-based one. At that step of the game, participants had a high degree of uncertainty about the colour of the covered blue brick (C). Based on the participants’ point of view, it could have been green, black, yellow, blue or orange because, at this point of the task, the last brick taken was green. The robot did not suggest that covered brick yet; thus, it could be the same colour as the last brick taken (or one of the colours with a lower value than green). Thus, to the brick C, which is the $\hat{x}$ of this conflict, the participants associated a uniform probability distribution over the colours listed above.

The shared perception model aims at minimising the participants’ uncertainty about the covered object’s characteristics. Following the model, the SP-robot indicated the blue brick visible to both agents (D). Indeed, the robot revealed that it could not perceive anything better than a blue brick: the covered brick C had necessarily a lower or equal value than the brick D: it could only be blue or orange. Pursuing the same uncertainty minimisation goal, if participants took D, iCub indicated the other blue brick (C) in the next move. Otherwise, if they took one of the yellow bricks (E-G), it indicated the visible blue (D) again. On the other hand, during NSP sessions, the robot decided only on its own knowledge and data structures, indicating at first the blue brick (C), which was hidden from the participants’ view. If participants took it, iCub then indicated the other blue brick (D); otherwise, if they took one of the yellow bricks (E-G) or the visible blue one (D), it then indicated the hidden brick (C) again.

##### 3.2.2.2 The First Brick Conflict

**FIGURE 4:**
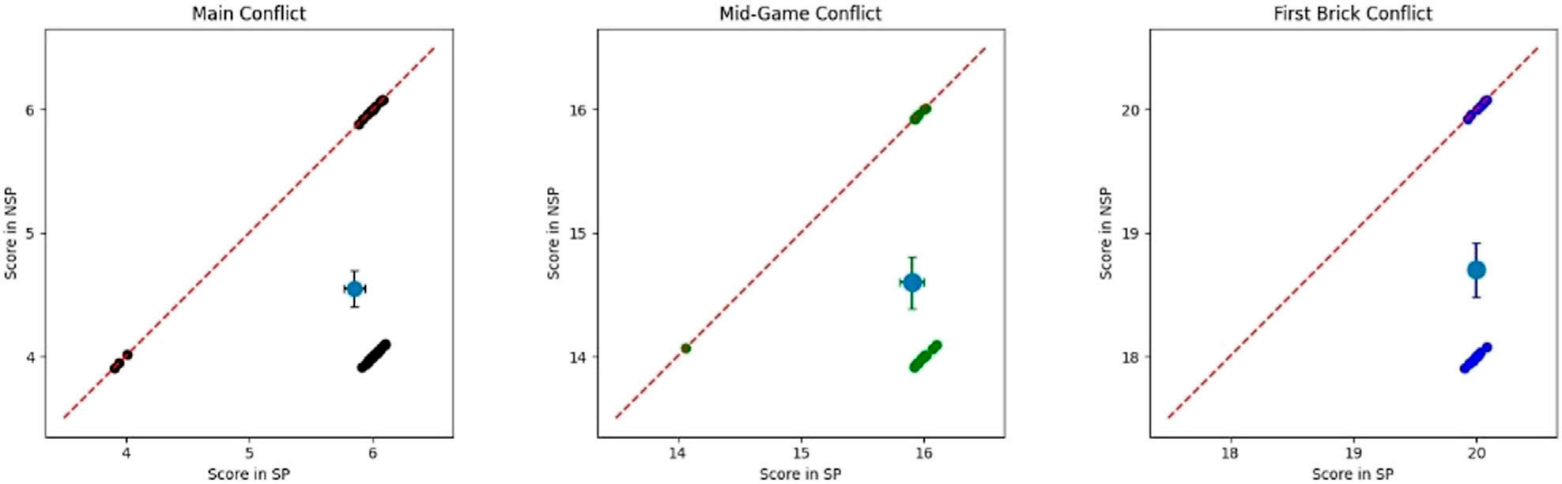
Participants’ score in both SP and NSP conditions during the conflicts. **(A)** is referred to the Main Conflict, **(B)** to the Mid-Game Conflict, and **(C)** to the First Brick Conflict. We applied a Gaussian random noise (*μ* = 0, *σ* = 0.05 on both the *x* and *y* axis) to make all of them visible. It is also shown the average score with standard error.

This conflict occurred at the beginning of sessions based on the first setup; this means that each participant faced twice this type of conflict. The bricks’ configuration related to this conflict is shown in Figure 4 (right). As we can see from the figure, the core of the conflict was the red brick covered to the participants but visible to the robot (F). The red colour corresponds to the highest value on the scale, representing one of the best choices to start a tower. At the beginning of the task, the brick F could be of any colour with the same probability. Thus, if we assume that the brick F was the $\hat{x}$ of this conflict, the participants associated a uniform probability distribution over all the colours reported on the list.

In the SP condition, the robot followed the model and selected the hint aimed at minimising the participants’ uncertainty about the covered object’s characteristics by leveraging on the characteristics of the objects visible to both the participant and itself. Hence iCub indicated it (F) at first to reveal that the covered brick F could not be worse than the visible red brick (A), which means that it could be only either red or white (corresponding to the same value). If participants took that brick in the first round, iCub then indicated the visible red brick (A); otherwise, if participants took at first the visible red brick (A), it then indicated the hidden brick (F) again. Instead, during the NSP sessions, the robot indicated the red brick visible to both (A) firstly and afterwards it indicated the hidden red brick (F).

##### 3.2.2.3 The Mid-game Conflict

**FIGURE 5:**
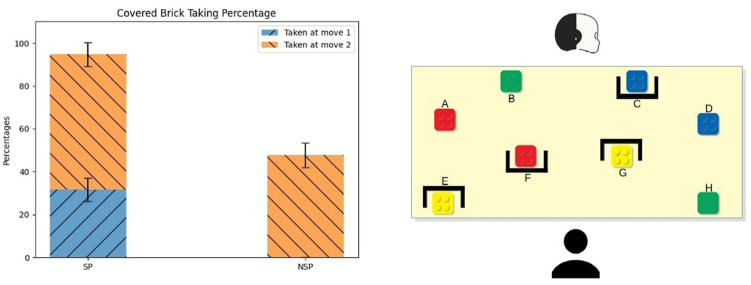
Average and standard error of the First Brick Conflict’s covered brick taking percentage. In this case, the covered brick was the F one.

This conflict occurred in the middle of game sessions based on the second setup; this means that each participant faced twice this type of conflict. An example of the bricks configuration during this conflict is shown in Figure 5 (right side). The core of the conflict was the green brick (M), hidden to the participant but visible to the robot. The colour green was associated with the highest value available on the table in that portion of the game. At this point of the game, participants had a high uncertainty about the colour of the covered green brick M: from the participants’ perspective, it could be red, white, green, black, yellow, blue or orange.

The robot, guided by the SP model, aimed to inform participants that there was something interesting that they could not see. Hence, iCub indicated at first the green brick (M) hidden to the partner. This way, the robot revealed that the covered brick M was equal to or better than the visible ones, which means that it could be either red, white or green. The robot minimised the participants’ uncertainty about the covered object’s characteristics through its communication signal. If participants took it, iCub then indicated the other green brick (N) visible to both; otherwise, if they took the visible green block (N) in the first round, iCub then indicated the hidden one (M) again. On the other hand, during NSP sessions, the robot indicated what looked best from its viewpoint, irrespective of the human point of view. In particular, it first indicated the green brick visible to both (N) and then the hidden green brick (M).

##### 3.2.2.4 Step-by-step Task Simulation

For clarity, we provide here a simulation step by step of a session with the robot in both conditions. For simplicity, in these simulations, we consider that the participants always follow the robot’s suggestions. We start from the SP-robot and the bricks setup 1 (Figure 2). First, we encounter the configuration of the *First Brick Conflict*. Thus, the robot would suggest the brick F. Due to the presence of the brick A in the robot and the participant’s shared perception; such a suggestion shows that the brick F (with a value of 10) is better (or no worse) than the brick A (with value 10) because otherwise, the robot would have suggested this latter. Then, the robot would suggest brick A, which is the best choice from its perspective. Then, it would suggest the bricks B and H (both with value 8) for the same reason. Finally, the configuration of the bricks becomes that of the *Main Conflict*. In this case, the robot would suggest the brick D: such a suggestion shows that the brick D (with a value of 4) is better (or no worse) than the brick C (with a value of 4), which is covered to the participant. This concludes the session. Now, we move to the NSP behaviour using the same setup. First, the robot would suggest brick A (with a value of 10), which is one of its best choice and the first brick according to its internal representation. Then, it would suggest the bricks F (with a value of 10 but covered to the participant), B, and H (both with a value of 8) in this order for the same reason. Lastly, the robot would suggest the brick C (with value 4), which is one of the best choices from its perspective and the first one according to its internal representation of the task but covered to the participant.

#### 3.2.3 Pilot Experiments

Before running the experiments with the robot, we performed a pilot study with eight colleagues in a human-human configuration. One participant took the role of suggester, while the other took the builder’s role. The pilot aimed to study the nature of the signals used by people in exploiting SP mechanisms. Before starting the task, we asked participants to use only nonverbal communication.

Through videos, we noted that all participants used gaze cues to indicate the objects: sometimes, a movement with the eyebrows followed the gazing. The suggesters tried to attract the other participant’s attention by establishing eye contact; then, they gazed at the candidate brick. At the end of this gazing exchange, the builders followed the suggesters’ hints. Sometimes, especially in the first trials, the builders asked for a confirmation by pointing or gazing at the object they wanted to take. The suggesters attempted to make the builders aware of their hidden bricks in every trial.

### 3.3 Participants

We had 22 participants (9 males and 13 females) with an average age of 26.5 years (SD: 7.8). Two participants failed to understand the experiment instructions (*i.e.*, did not choose the highest valued brick as the first element of a tower) and were therefore discarded from the analysis. All participants gave written informed consent before participating and received a fixed refund of *£*15. The experimental protocol was approved by Regione Liguria’s regional ethic committee.

### 3.4 Measures

During the experiment, we collected some behavioural measures such as the participants’ score, the number of times participants followed the robot’s hints, the time needed to take a brick (calculated as the time between the beginning of the action phase and the grip of the brick), and what bricks the robot indicated. In particular, we focused on the conflicts where the mechanism of shared perception could have had an impact.

#### 3.4.1 Questionnaires

We submitted questionnaires to participants before the beginning of the experiment and after each interactive session with the robot. Before the experiment, we asked participants to reply to the Seventeen-Item Scale for Robotic Needs (SISRN) questionnaire (Manzi et al., 2021). We chose the SISRN questionnaire to know what participants thought about generic robots’ capabilities. Furthermore, both before the experiment and after each experimental session, we submitted to participants the Godspeed (Bartneck et al., 2009) and the Inclusion of Other in the Self (IOS) questionnaires (Aron et al., 1992). The questionnaire web page contained a video of the iCub robot^2^: we showed it to participants to provide them with enough information about the robot before a real interaction with it. The Godspeed questionnaire was chosen to collect participants’ impressions about robot’s *anthropomorphism*, *animacy*, *likeability*, and *perceived intelligence* before and after the interactive sessions. The IOS questionnaire was used to understand if participants felt closer to the robot in some of the two experimental conditions.

## 4 Results

The collaborative game with the iCub robot was characterised by perceptual asymmetries between the human-builder and the robot-suggester. This asymmetry was particularly critical in certain choices during the game (the *conflicts*, see Section 3), where the best brick to take could differ between the two agents’ perspectives. We focus our analysis on these specific moments in the game: the *Main Conflict* (Section 3.2.2.1), the *First Brick Conflict* (Section 3.2.2.2) and the *Mid-Game Conflict* (Section 3.2.2.3), to assess the impact of the robot’s suggestions to the partner, when they are based on a shared perception mechanism (SP) or not (NSP). The former considers the brick visibility to the human in selecting which brick to suggest, whereas the NSP-robot just relies on its internal representation of the task.

First, we checked whether the configuration of the bricks affected participants’ performances. For this purpose, we split the total scores obtained in each bricks’ configuration for each robot mode. We conducted paired t-tests and we found no significant differences between the scores obtained with the two setups, with the robot in SP mode (*paired t-test* *t* (19) = −0.567, *p* = 0.578); and in NSP mode (*paired t-test* *t* (19) = 0.837, *p* = 0.413)).

### 4.1 The Main Conflict

Figure 3 (left) shows the average percentage of time participants picked one of the yellow bricks (either E or G)—covered to the robot’s sight. These corresponded to the best choice for the participant, as these bricks had the highest value. During the SP sessions, more than 80% of the participants took a yellow brick; conversely, only 40% of the participants took one of the current best bricks during NSP sessions (*μ*_*sp*_ = 84.21, *SE*_*sp*_ = 5.99; *μ*_*nsp*_ = 38.09, *SE*_*nsp*_ = 7.58). Instead, they took the brick iCub was indicating them: the blue brick C. The difference between the two conditions is significant (*two-tailed z-test,* *z* = 2.21, *p* = 0.02).

There was no difference in the time employed to pick the brick in the two conditions. The timing was computed only for participants who picked the covered block, hence on the percentages of participants reported in Figure 3. Figure 4A shows the scores of participants collected during the Main Conflict in both the experimental conditions. As we can see from the plot, around 40% of the participant scored more during SP sessions than during the NSP ones.

### 4.2 The First Brick Conflict

Figure 5 (left side) shows the percentage of times in which participants picked the brick covered to them (F). As we can see from the figure, during the SP sessions, participants resolved the conflict properly almost all the time: more than 30% of the time took the brick F as their first move (*μ*_*sp*_ = 31.57, *SE*_*sp*_ = 5.4), and around 65% of the time took it as their second move (*μ*_*sp*_ = 63.15, *SE*_*sp*_ = 5.6). The remaining 5% of the time, participants did not take the brick F; instead, they preferred to take a green brick. All the participants who did not take the brick F firstly during the SP sessions took the brick A as their first move. On the other hand, during the NSP sessions, nearly 50% of the time, participants could take the brick F (*μ*_*nsp*_ = 47.61, *SE*_*nsp*_ = 5.7) While the remaining have opted to take a green one. All participants took the brick A as their first move during the NSP sessions. The difference between the two conditions is significant (*two-tailed z-test*, *z* = 3.81, *p* < 0.001), while the difference between the percentage referred to the covered brick taken at move two was not.

Also, there were no significant differences in the time employed to pick the hidden brick between conditions for this conflict. Figure 4C shows the scores participants collected during the First Brick Conflict in both the experimental conditions. As we can see from the plot, around 50% of the participant scored more during SP sessions than during the NSP ones.

### 4.3 The Mid-game Conflict

**FIGURE 6:**
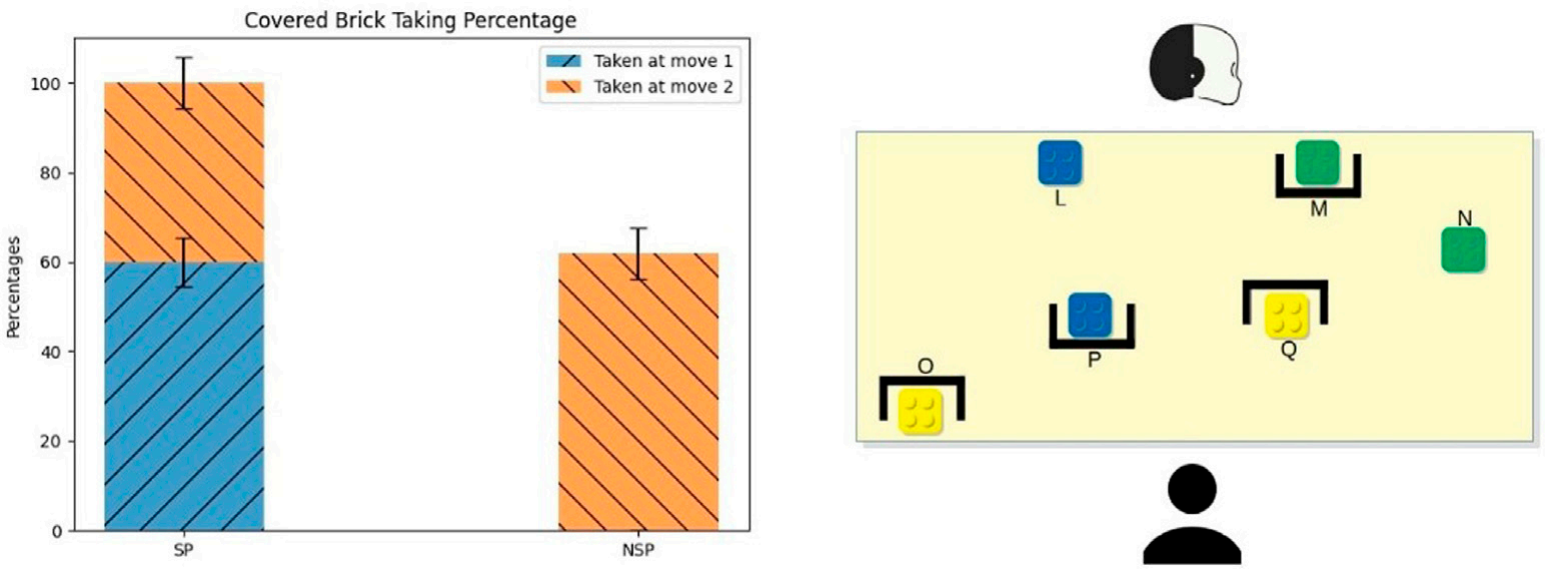
Average and standard error of the Mid-Game Conflict’s covered brick taking percentage. In this case, the covered brick was the M one.

Figure 6 shows the percentage of times in which participants took the covered green brick M. Participants behaved quite the same as during the previous conflict: during the SP sessions, 60% of the time, participants took the brick M as their first move (*μ*_*sp*_ = 60, *SE*_*sp*_ = 5.4), and the remaining 40% of the time they took it in the next move (*μ*_*sp*_ = 40, *SE*_*sp*_ = 5.6). Thus, all participants could resolve the *Mid-Game Conflict* properly during the SP sessions. On the other hand, during the NSP sessions, only 60% of the time, participants could take the brick M (*μ*_*nsp*_ = 61.9, *SE*_*nsp*_ = 5.7), while the remaining 40% opted to take a yellow one. As happened in the previous conflict, all participants took the brick N as their first move during the NSP sessions. The difference between the two conditions is significant (*two-tailed z-test*, *z* = 4.03, *p* < 0.001), while the difference between the percentage referred to the covered brick taken at move two was not significant.

Also in this case, the time employed to pick the hidden brick did not differ between conditions. Figure 4B shows the scores participants collected during the Mid-Game Conflict in both the experimental conditions. As we can see from the plot, around 40% of the participant scored more during SP sessions than during the NSP ones.

### 4.4 Questionnaires

**FIGURE 7:**
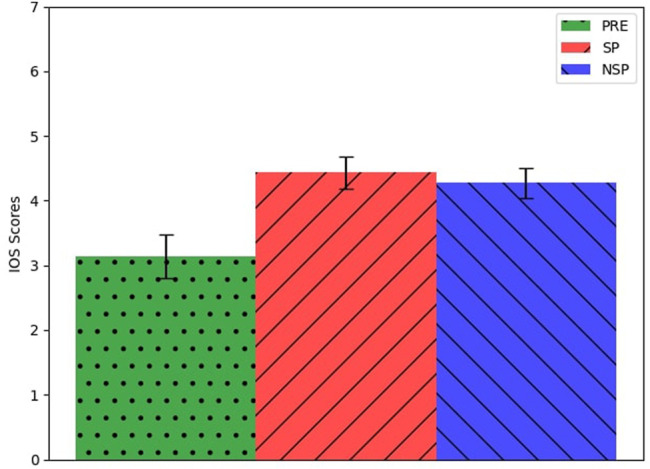
Average and standard error of participants’ answers to the IOS image.

Figure 7 shows the average and the standard error of their answers to the IOS image. As we can see, there is a difference between the answers given before the experiment and the ones given after the interactive sessions, regardless of the robot mode. These difference turned out to be statistically significant for both PRE-SP and PRE-NSP groups (*repeated measures ANOVA* *F* (21) = 6.82, *p* = 0.01 and *F* (21) = 6.6, *p* = 0.01, respectively). Nonetheless, we found no significant differences between the answers given after the SP sessions and those given after the NSP sessions.

**FIGURE 8:**
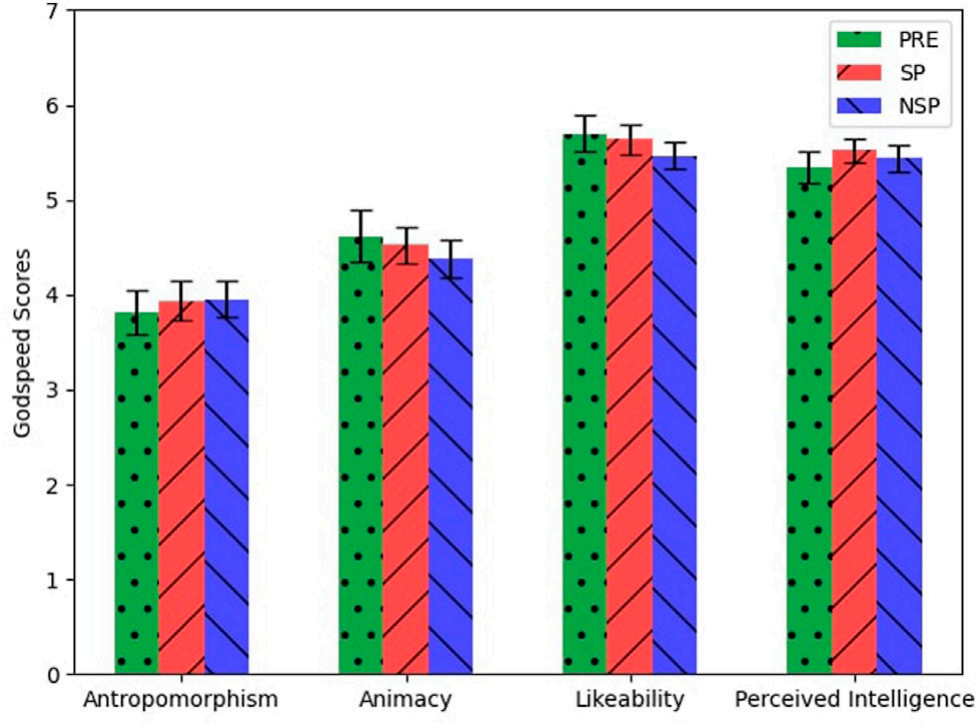
Average and standard error of participants’ answers to the Godspeed questionnaire.

Similarly, there were no significant differences in the Godspeed questionnaire (Figure 8) scores among the different phases (PRE, POST-SP, POST-NSP). We found no correlations between the answers given to the SISRN and the other questionnaires or the participants’ performance during the conflicts.

## 5 Discussion

In this work, we assessed whether a robot attempting to establish a shared perception with its human partners is better evaluated and ensures a more effective collaboration. The results suggested that shared perception leads to higher performances in the task. A robot considerate of the partners’ viewpoints and goals can facilitate selecting the best action.

The proposed mathematical model for shared perception, which guided iCub selection of the hints to be provided to the partner in the SP condition, seemed to be effective. The robot indicated with its gaze the brick that minimised participants’ uncertainty about the properties of the objects hidden from their view. This did not imply that the players picked the suggested object right away. Instead, it successfully ensured that participants took into account also the covered bricks in their reasoning, thanks to the implicit knowledge shared by the robot. As a result, in the SP conditions, the vast majority of the time, participants did not miss any of the highest value elements while building their Lego tower.

We hypothesised that participants would use a recursive (or second-order) Theory of Mind (ToM) when reasoning about the robot’s suggestions: e.g., participants knew that the robot knew that they could not perceive the bricks covered to them (for example, the brick F in Figure 2A). Indeed, several models have been presented that make use of recursive ToM (Pynadath and Marsella, 2005; Bosse et al., 2011; de Weerd et al., 2017). In particular, Bosse et al. (2011) presented a model for multilevel ToM based on BDI concepts that they tested in three different case studies: social manipulation, predators’ behaviour, and emergent soap stories. Instead, Pynadath and Marsella, (2005) proposed PsychSim, a multi-agent simulation tool for modelling interaction and influence that makes use of a recursive model of other agents. Moreover, de Weerd et al. (2017) proved that agents using second-order ToM lead to higher effectiveness than agents capable of only first-order ToM. Moreover, more importantly for us, they discovered that people spontaneously use recursive ToM when their partner is capable of second-order ToM as well.

Instead, without shared perception, the robot hints were less informative. It followed the rationale of indicating the highest valued brick from its perspective. In particular, the robot relied only on its internal representation of the task, which led it to behave during conflicts in the opposite way to the SP setting. Thus, we ensured the maximum difference between the SP and NSP behaviours. This led to errors, in particular when the best bricks were not visible to the robot (*Main Conflict*). However, also in situations in which the asymmetry in perception was not so critical (*First-Brick* and *Mid-Game* conflicts), as the best blocks were hidden to participants but not to the robot, its hints were less effective. A significantly lower percentage of players gathered all the best bricks in the NSP condition than in the SP.

We speculate that during SP sessions, participants built a more precise representation of the robot’s perspective than during NSP sessions. In the former sessions, they better understood that when the robot was indicating a brick visible to all, it was because it had nothing better to suggest. Consequently, they could resolve the conflict correctly and pick the best option, even when they could not see it directly. In NSP sessions, the participants did not have enough information to understand the reasons guiding the robot suggestion and gauge their validity. As a result, they blindly followed the robot’s hints in some cases. In particular, in 50% of the cases in the *First Brick conflict* and 60% of those in the *Mid-Game conflict*, participants’ second move followed iCub’s indication toward the covered object even if there was very limited information about what brick the robot was indicating to them. In those conflicts, this choice was still valid, as the suggested brick had a value as high as the visible ones. In the *Main conflict* instead, the excessive trust led in about 60% of the cases to a sub-optimal choice. In other cases, the lack of understanding of the robot’s motives led participants to disregard the robots’ suggestions, missing out on valuable blocks hidden from participants’ view. Indeed, about 40%–50% of the time, participants in one of the conflicts went on picking visible blocks, whereas the robot was pointing at the highest one behind an occlusion. This means that the absence of a reliable common ground makes people unable to fully exploit the collaboration.

An interesting reflection about participants’ trust toward the robot can be afforded by the *First Brick* task configuration. In this conflict, participants’ choice could not be driven by the outcome of previous moves, as it regarded the first move of a game. Furthermore, the games previously played with the robot should not have had any influence since, at the beginning of each session, the experimenter instructed the participants that a new program controlled the robot. In this case, for the game’s first move, two bricks of the highest possible value are present on the scene, one visible and one hidden from the participants’ view. Since there were no bricks with higher value in the game, the most rational first choice would have been picking the visible highest value brick. Despite this, when the robot indicated the hidden item (i.e., in the SP condition) in around 30% of cases, participants opted to pick that instead. Overall, this result suggests that for a good portion of participants, the robot indication was sufficient to make them abandon a sure optimal choice, to pick something unknown. We ascribe the choice to participants’ proneness to trust (or better over-trust) the robot, a phenomenon often observed in interactions with robots. An alternative explanation is that this choice was driven by an attempt to behave kindly toward the humanoid. Recent evidence points out that also in HRI, mechanisms like reciprocity play a role—with humans overtly following the robot’s advice despite disagreeing with it, to ensure its future benevolence, as it happens between humans (Zonca et al., 2021b).

It is also relevant to notice that in the experiment, there was also an analogous configuration in which the iCub indicated first the visible highest value brick and then the hidden highest value brick (i.e., in the NSP condition). In this case, the proportion of participants who followed the second indication and managed to pick also the hidden high-value item reached about 60% of the cases. Although higher, this implies that in about 40% of the situations, seeing that the robot indications were meaningful in the first move was not enough to induce participants to trust its indications in the next move. In other words, the fact that iCub indicated the best brick among those the participants could perceive did not convince participants to select the item it indicated when it was not visible. Such a lack of trust led those participants to miss a relevant opportunity. Apparently, the less trusting participants did not receive enough information about what drove the robot’s suggestions to follow them. These findings underline the importance of the robot selecting its hints properly by revealing as much as possible to the partner its own understanding of the environment. By doing so, the robot can avoid, on the one hand, over-trust and the other excessive lack of trust.

Despite the difference in overall performances between SP and NSP sessions, we registered no differences between the answers to the post-session questionnaires. This contradiction shows us SP mechanisms’ essential and implicit nature: people exploited SP mechanisms, but they were not fully aware of them. In fact, participants reported no particular differences between the robot’s behaviours according to the different experimental behaviours.

In our experiments, we defined two robot behaviours that resulted in being at the antipodes, with the NSP robot actually being a *anti-*SP mode. A fairer NSP behaviour would provide randomised choices from the robot. However, such a less controlled design (e.g., with a robot selecting randomly in case of conflict in the NSP condition) would have required a much larger sample to enable reliable testing of all the possible conditions. To explore this option, we ran a series of simulations.

More precisely, we conducted 1,000 simulations in which we tested a fair NSP robot mode using the results of users’ behaviour that we obtained in our experiments. In such simulations, in case of multiple best options, we let the NSP robot choose randomly between them. On the other hand, we defined the simulated users’ behaviour based on the experimental results. This means that, in NSP-mode, when the hidden brick is suggested as a second move, users resolve the conflict 50% of the time during the First-Brick conflict, 60% during the Mid-Game conflict, and 40% during the Main conflict. The same applies for the SP-mode: 100% during the Mid-Game conflict, 95% during the First-Brick conflict and 83% during the Main conflict. Then, we analysed how many times the simulated users resolved the conflicts. Table 2 shows the statistics about the resolution of such simulated conflicts. As we can see, we obtained results comparable with those derived from the real experiments.

**TABLE 2:** Results of the simulation of the experiment with the fair NSP robot behaviour. The statistics are applied on the simulated data.

	% of Resolution		*Two-tailed z-test*	
	SP	NSP	*z*	*p*
Main Conflict	80.2	64.3	8.97	<0.001
First-Brick Conflict	90.8	71.4	11.08	<0.001
Mid-Game Conflict	100	59	22.7	<0.001

In this work, we considered a simplified scenario capturing the main elements of intriguing HRI collaborative situations: asymmetries in perception and objects having properties that make them more or less suitable to be used next. To focus on these central aspects, we opted for simplifying the settings: objects have a single relevant property (their colour); and the communication is minimised: we allowed only gazing to indicate the proposed object. However, in principle, the model we propose could be generalised to more complex settings, as far as the robot can 1) estimate the impact of its suggestions on the uncertainty of the partner’s representation, 2) have a measure of relative (to the task) importance to assign to each object’s characteristic, and 3) have richer communication capabilities. We refer the reader to Section 5.1, where we better discuss what a more generalised approach could concern. In fact, we can easily map our experimental task into the more complex assembly task that we give as an example in Section 1. As long as the person and the robot are aligned on the assembly step, the robot can use our model to choose what to say or which object to pass in order to maximise the flow of information. Let us imagine a scenario in which two sets of screws are appropriate for the assembly, one well visible to both the robot and the participant and one partially occluded to the latter. Through our model, the robot can decide it is worth suggesting or handing over the screws from the semi-occluded set to maximise both performance and the person’s knowledge about the available tools in the chaotic environment. This way, the robot can lower the person’s uncertainty about objects not included—or partially included—in their perception.

The scenario in which humans and robots have a misaligned perception of the shared environment has been addressed by Chai et al., 2014. Their work focused on allowing a robot to acquire knowledge about common ground via collaborative dialogue with its human partner: the more the communication proceeds, the more the robot can improve its internal representation of the shared environment. The main aim of their approach was to help the robot in lowering its uncertainty about semi-occluded objects. Our work addresses the task proposed by Chai et al., 2014 from the opposite perspective by proposing the robot as a suggester, helping the human in resolving asymmetries in the shared environment. Moreover, we explored interactions that do not involve the use of speech. We addressed the shared perception problem using more primitive communicative ways, thus without considering the language.

To conclude, it is essential to note that a direct prediction of our model is that if two agents share nothing in their common awareness spaces, then it is impossible to obtain shared perception. Hence, we can claim that establishing common ground is key to pursuing a collaborative HRI task. Thus, it becomes crucial to building shared knowledge about both the environment—which can present asymmetries in perception, as in our experiments—and the collaborating agents’ mental states in terms of objectives, beliefs and intentions.

### 5.1 Limitations

The first limitation of our work regards the simplifications we made in our experimental setting. In particular, the interaction was constrained during the experiments, and only nonverbal communication was allowed. We allowed only a turn-based speechless communication to maintain careful control of the information exchange with the participants and to ensure that all participants faced the “conflict” instances with the same amount of information. This is obviously a simplification: communication between partners is usually more complicated than this. However, we believe that there are forms of real interactions which are not too far from the settings we proposed, such as some turn-based card games (e.g., “Briscola” we mentioned above) and assembling tasks, where the context constrains the interaction.

Other limitations regard some assumptions of the model. In particular, the model assumes that the objects’ features do not change over time. This limitation can be overcome by introducing memory-based and/or probabilistic measures of uncertainty regarding the objects and their characteristics. In particular, for what regards the object the robot is aware of but that not perceive anymore, such a measure of uncertainty could model how stronger the robot believes the object is still where it remembers (e.g., a measure that could worsen over time). A similar argument could be applied to mutable characteristics of particular objects. A fluid measure of uncertainty can manage how much the robot is sure about an object’s characteristics (e.g., the shape of a partially-occluded object could be challenging to understand but easily guessable).

Furthermore, the robot already knew which bricks the participants could perceive and which ones they could not. To make the architecture more autonomous, we could use perspective-taking to allow the robot to automatically infer the objects belonging to the partner’s personal perception (Fischer and Demiris, 2020). Also occlusions (the obstacles in our experiment) could be detected through perspective-taking algorithms.

Finally, we assumed that participants would accept the suggestions the robot gave as the bests to achieve the goal of the task. Before starting the experiment, we presented the robot as a collaborator but, in general, we should take into account the level of trust people have towards robots (Zonca et al., 2021a).

## 6 Conclusion

We investigated the role of Shared Perception (SP) in Human-Robot Interaction (HRI). In particular, with the present work, we aimed to 1) understand whether and how humans would exploit SP mechanisms with a robot during a cooperative game characterised by an asymmetry in the perception of the environment and 2) propose a computational model for SP. Indeed, we designed a mathematical model for cooperative SP. We tested it via a user study in which the robot and participants had to collaborate to build a tower with LEGO bricks. Some of those were visible by both agents, others were covered to the participants, and the remaining were covered to the robot. We designed our experiment to elicit critical moments that we called *conflicts*, and we investigated the differences between a robot with SP (SP-iCub) and a robot unable to use SP mechanisms (NSP-iCub) when the perceptions of the interacting agents differed.

Our results show that humans can potentially exploit SP mechanisms with robots as they do with other humans. For all conflicts, SP-iCub resulted to be more informative than NSP-iCub. Indeed, with the former, people could correctly resolve conflicts most of the time. Conversely, only a minority of the participants could make the best move in such critical situations with the latter. However, despite the clear difference between the experimental conditions and the resulting strategies that we registered, our participants did not report perceiving the robot’s behaviours differently. This effect highlights the implicit nature of SP: people exploit SP mechanisms but are unaware of their decision process.

## Data Availability

The raw data supporting the conclusion of this article will be made available by the authors, without undue reservation.

## Footnotes

1
https://en.wikipedia.org/wiki/Briscola.

2
https://www.youtube.com/watch?v=3N1oCMwtz8w.
